# My joint pain, a web-based resource, effects on education and quality of care at 24 months

**DOI:** 10.1186/s12891-020-3074-2

**Published:** 2020-02-06

**Authors:** Xia Wang, Hema Urban, Kim L. Bennell, Chris Dickson, Fiona Dobson, Marlene Fransen, Graeme Jones, David J. Hunter

**Affiliations:** 1 Institute of Bone and Joint Research, The Kolling Institute, University of Sydney, Royal North Shore Hospital, Reserve Road, Level 10, Kolling Building, St. Leonards, Sydney, NSW 2065 Australia; 2 Centre for Health, Exercise and Sports Medicine, Department of Physiotherapy, University of Melbourne, Melbourne, Victoria Australia; 3 Arthritis Australia, Glebe, New South Wales Australia; 4 Discipline of Physiotherapy, Clinical and Rehabilitation Sciences Research Group, Faculty of Health Sciences, University of Sydney, Lidcombe, New South Wales Australia; 5 Menzies Institute for Medical Research, University of Tasmania, Hobart, Tasmania Australia; 6 Rheumatology Department, Royal North Shore Hospital, Sydney, New South Wales Australia

**Keywords:** Quality of care, Digital health, eHealth, Self-care, Osteoarthritis, Internet interventions

## Abstract

**Objective:**

To evaluate the effects of the updated version of an evidence-based osteoarthritis (OA) resource and consumer hub, ‘My Joint Pain’ website, on health education and quality of care over 12 months.

**Methods:**

Using a classic quasi-experimental design, participants with symptomatic hip or knee OA were recruited across Australia to evaluate the ‘My Joint Pain’ website, compared to a control group of non-users from 12 to 24 months. Outcome measures included the Health Education Impact Questionnaire (HEIQ) and the OA Quality Indicator (OAQI) questionnaire. The changes from 12 to 24 months in the HEIQ were evaluated using a generalised linear model. The differences between users and non-users in the OAQI were evaluated using a chi-square test.

**Results:**

A total of 277 eligible participants with symptomatic hip or knee OA were recruited at baseline, and 122 participants completed the 24-month surveys (users: *n* = 35, non-users: *n* = 87). There was no significant difference between users and non-users for the HEIQ scores at 24 months after adjustments for age, sex and body mass index (BMI). Users had higher emotional distress scores than non-users in univariable analysis. When compared with non-users in the OAQI, users showed favourable changes in receiving information about “self-management” and “acetaminophen” and “non-steroidal anti-inflammatory drugs (NSAIDs)” from 12 to 24 months.

**Conclusion:**

The evaluation of the updated ‘My Joint Pain’ website didn’t find significant improvements in terms of health education, but it may help delivering useful information about self-management and appropriate use of pharmacological treatments. More strategies are needed to facilitate the uptake of evidence-based self-management and education online resources for OA consumers.

## Introduction

Osteoarthritis (OA) is a leading cause of chronic pain and one of the top contributors to global disability [1]. As the most common form of arthritis, OA currently affects 2.1 million (9%) Australians of all ages. Particularly, in people over the age of 45, the prevalence increases to 21% [2]. In addition to the individual burden of OA, the direct and indirect costs also contribute to a substantial socioeconomic cost [3]. By 2020, it is estimated that the prevalence of OA will increase by 50% due to an ageing and increasingly obese population [4].

Evidence-based clinical guidelines recommended non-surgical, non-pharmacological treatments, such as exercise, weight loss and self-management education, as the cornerstone for the care of OA [5–8]. However, current clinical practice is still largely limited to analgesic treatment followed by eventual joint replacement surgery [9]. The underuse of recommended management strategies and low levels of referrals to allied health professionals who can provide lifestyle interventions have been frequently reported within Australia and globally [10, 11]. In fact, 81% of patients indicated they would not accept surgery if alternative treatments were offered, and physiotherapy was the most popular option [12]. Additionally, people with OA often lack enough knowledge of their condition and have limited involvement in clinical decision making [13]. The provision of evidence-based information to patients outside the clinical encounter has long been carried out aiming to increase their self-efficacy and health outcomes. These included providing structured education and additional personalised support, allowing patients to play an active role in improving their condition [14, 15]. However, current OA self-management education programs have small or no clinical benefits and do not improve patient’s self-management skills [15]. It is suggested that investigations of alternative models of delivery for those programs may be warranted [15].

In recent years, the use of the Internet as a source of health information to aid the management of chronic diseases has become increasingly popular across diverse socioeconomic and age groups [16]. With the rising disease burden of an ageing population on the overstretched healthcare system, more people accept Internet-based health solutions as part of their management, especially if it facilitates their personal care [17]. Compared to in-person interventions, digital health interventions are more cost-efficient and can reach a broader population in remote areas [18]. It allows people with chronic conditions to administer their treatment at a suitable time point which could increase treatment adherence and compliance, ultimately, improving their health and quality of life. However, Internet-based health resources still have many obstacles which limited their medical usage. For example, much online information often failed to incorporate with up-to-date research evidence [19]. An investigation of 37 unique websites found the readability and quality of online health information for OA was more difficult than the recommended level defined by the Journal of the American Medical Association benchmark criteria [20]. Despite the increasing utilisations of Internet-based self-management among people with chronic diseases, only a few studies have targeted people with OA [21, 22]. These studies provide preliminary evidence of the effectiveness, acceptability and feasibility of different Internet-delivered self-management interventions, but long-term data is scarce.

To this end, we have previously investigated the effect of a publicly available resource, the ‘My Joint Pain’ website, which contains evidence-based OA management resources and self-management tools aiming to empower users in the clinical encounter and informed decision-making [23]. In a 12-month evaluation study, we found patients who used the website had significant improvements in several health education domains including health-directed activity, positive and active engagement in life, self-monitoring and insights, skill and technique acquisition, and social integration. Improvements in important aspects of quality of care, such as self-management, lifestyle and weight reduction, were also observed in people used the website [23]. Although the website provides extensive information on evidence-based treatments and facts about the disease, appropriate psychological approaches and technological strategies are still insufficient to assist the delivery of that information and shift participants’ viewpoint about the impact of OA [23]. In regard to health service navigation, the website does provide a tool to locate nearby healthcare resources. However, this tool is not easily accessible, well-integrated, and not located on the main toolbar, which might account for the observed outcome [23]. In response to our findings, the website has been redesigned to address the gaps highlighted by the results. The current study aims to investigate further the effects of the updated website on improving consumers’ health education and quality of care over 12 months.

## Methods

### Study design

Using a quasi-experimental study design, the ‘My Joint Pain’ website was evaluated by comparing a group of users of the website and a group of non-users in a population afflicted with OA of the hip and/or knee. Responses to the outcome measures were collected at baseline, 12 months and 24 months. Outcomes from the 12-month evaluation have been previously published and influenced modification to the resource that was re-evaluated at 24 months to investigate before and after changes between groups [23]. This study was approved by the Human Research Ethics Committee of the Universities of Sydney (no. 2014/017) with all participants providing informed consent online.

### Participants

Participants across Australia were invited to take part in this study through advertisements and dissemination by Arthritis Australia, Melbourne Physiotherapy Department, Centre for Health, Exercise and Sports Medicine and the University of Sydney in 2014 [23]. Interested participants were directed to a screening questionnaire to identify symptomatic OA which consisted of 5 questions asking the participants if they had hip or knee joint pain in the past year, joint crepitus (grinding or clicking) during movement, joint pain when squatting and stiffness that lasted less than 20 min. Participants with symptomatic knee or hip OA was defined based on the America College of Rheumatology knee [24, 25] and hip [26] OA diagnostic criteria, which are having knee or hip pain and at least one of symptoms (i.e. crepitus, squatting pain, stiffness) from the screening questions. Participants were asked about whether they have been diagnosed as having hip or knee OA by a doctor. Eligible participants were aged ≥50 years old and had symptomatic hip/knee OA, or diagnosed by a doctor of hip or knee OA; had access to the Internet and an email address for communications.

### Intervention

The ‘My Joint Pain’ website was developed by Arthritis Australia (a charitable not-for-profit organization and Australia’s peak arthritis body) in collaboration with the BUPA Health Foundation (a not-for-profit organisation dedicated to health in Australia) and informed by an expert content committee comprising of leading OA researchers, clinicians and consumers [23]. The framework and content development of the website was based on several quality standards including guidelines for patient decision aids by the International Patient Decision Aid Standards (IPDAS) Collaboration [26], Australia’s National Health and Medical Research Council (NHMRC) Guidelines for consumer information and the Health on the Net (HON) code standard [27].

**Fig. 1:**
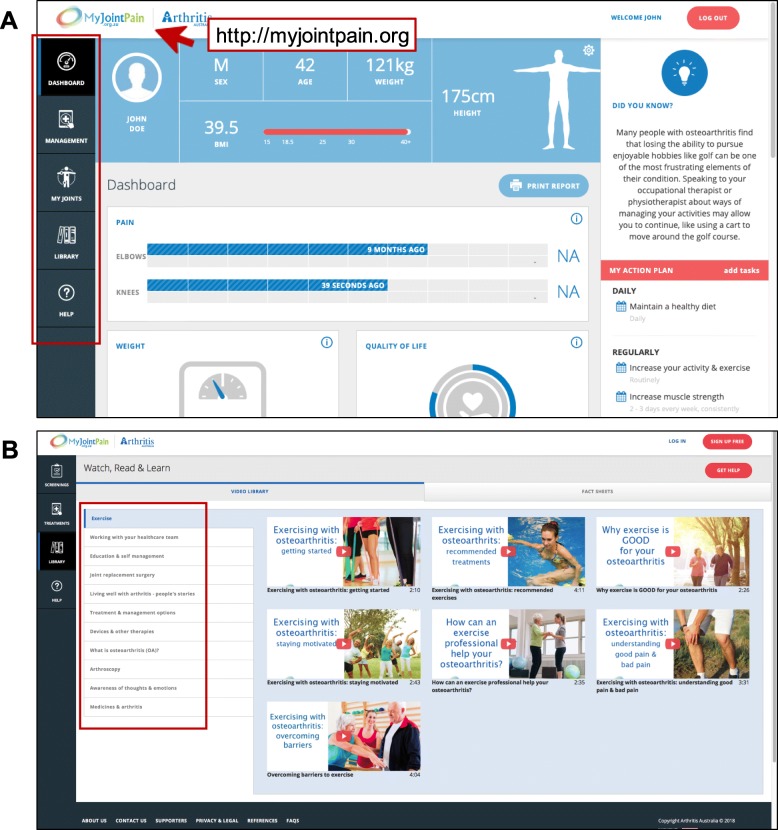
The updated ‘My Joint Pain’ website user interface. **a** The left custom menu to highlight the 5 key sections on the site (red rectangle). The dashboard shows participant’s most up-to-date profile including basic demographics and pain trajectory. The right menu includes osteoarthritis tips and “My Action Plan” which was customised for the participants according to their goals and tasks. The web address is embedded in the logo (red arrow) to help people finds it easily. **b** In the video library tab, videos were categorised by 10 themes that are related to different osteoarthritis, treatment options and patient stories (red rectangle)

After the 12 months follow-up, the ‘My Joint Pain’ website was re-developed to include feedback received from the 12-month evaluation and telephone interviews with users of the new website. Changes to the site included a new user interface and an updated menu with the following sections (Fig. 1):

1. Dashboard – A summary of current user information such as age, gender, BMI, pain severity and quality of life, medications and treatments.
2. Management – This page includes a further three tabs that include recommended treatments, progress tracking and a treatment action plan
3. My Joints – A page with weekly check-ups to collect and track patient information about levels of pain on each affected joint, current weight, received medications and treatments, comorbidities and quality of life.
4. Library – An extensive video and fact sheet library that includes educational information about lifestyle changes, self-management strategies, medications and surgical management in OA based on best available evidence, leading experts experience and consumer’s stories. The fact sheets are specially designed for OA of 11 joints, including hip, knee, hand, wrists, neck, feet, elbow, ankle, shoulder and back, and available in 12 languages such as English, Arabic, Chinese, Greek, Italian, Vietnamese, Croatian, Korean, Macedonian, Persian and Spanish.
5. Help – A page with frequently asked questions, contact information and health care providers information.

### Procedure

Eligible participants were asked to complete the baseline questionnaires including basic demographics, outcome measures and health services utilisation and were followed up at 12 months. Responders at 12 months were notified about the website updates and were able to choose to use the new website regardless of their previous usage. At 24 months follow-up, participants who indicated ‘yes’ to the question ‘Have you visited the ‘My Joint Pain’ website in the last 2 months?’ were classified as users and all other respondents were classified as non-users.

### Outcome measures

Two validated questionaries, Health Evaluation Impact Questionnaire (HEIQ) and the Osteoarthritis Quality Indicator (OAQI) were used to collect outcome measures at baseline, 12-month and 24-month follow-ups. The HEIQ is an instrument used to investigate the health education and psychosocial impacts of health education or self-management programs [28]. The 40-item questionnaire was used to evaluate the following 8 independent domains: 1) health-directed activities (4 items); 2) positive and active engagement in life (5 items); 3) emotional distress (6 items); 4) self-monitoring and insights (6 items); 5) constructive attitudes and approaches (5 items); 6) skill and technique acquisition (4 items); 7) social integration and support (5 items); 8) health service navigation (5 items). This uses a 4-point Likert Scale ranging from strongly disagree (score = 1) to strongly agree (score = 4) for each item. The final score for each domain was then calculated by summing the item responses and dividing by the number of items. All items in the evaluation were asked in the positive except emotional distress for which lower scores indicated a more favourable outcome. The difference between the means at the follow-up compared to baseline provided the change score.

The OAQI is a 17-item tool that evaluates the appropriateness of care received by investigating the information received by participants [29]. The instrument includes 6 questions that addressed patient education and information about disease development, treatment alternatives, self-management, lifestyle changes, weight management, and physical activity. Regular provider assessments were addressed in 4 questions. Four questions were related to pharmacologic treatment, and 3 addressed different referrals. The use of this instrument allows some understanding about barriers in information transfer and change in knowledge acquisition and outcomes were represented as a pass rate for the group. Each of the OAQI items was scored using a “Yes” or “No” format with a third option for “Don’t remember” or “Not applicable” items (i.e. “Not overweight” for the item on weight management). Each QI was considered eligible if the participant checked “Yes” or “No”. QI pass rates were tabulated as a percentage of “Yes” responses out of the total number of eligible responses. The changes in OAQI from the baseline to follow-up were categorised to as negative change, no change, or positive change (improvement) to indicate the change in the received care.

### Statistical analyses

As previously described, the evaluation of the ‘My Joint Pain’ website was carried out as part of a broader investigation beyond the targeted recruitment number in this study. Thus, 300 participants were invited to take part in this study [23]. The HEIQ was used to calculate the sample size with prior data indicating the difference in response of two groups to be normally distributed with a standard deviation of 1.2. In order to detect a true difference in the mean response of 0.6, we needed 44 participants in each group to have a power of 0.9 at a significance level of 95% [23].

The within-group changes for the HEIQ were evaluated using the pairwise analyses in a repeated measures model comparing 24-month outcomes to 12-month outcomes. A generalised linear regression model was used to compare users and non-users at 12 and 24 months adjusting for age, sex and BMI. Chi-square tests were used to evaluate the proportions of the improvement of quality of care using OAQI between the study groups. All statistical analyses were performed using STATA 15.0 for Mac (College Station, TX: StataCorp LP).

## Results

**Fig. 2:**
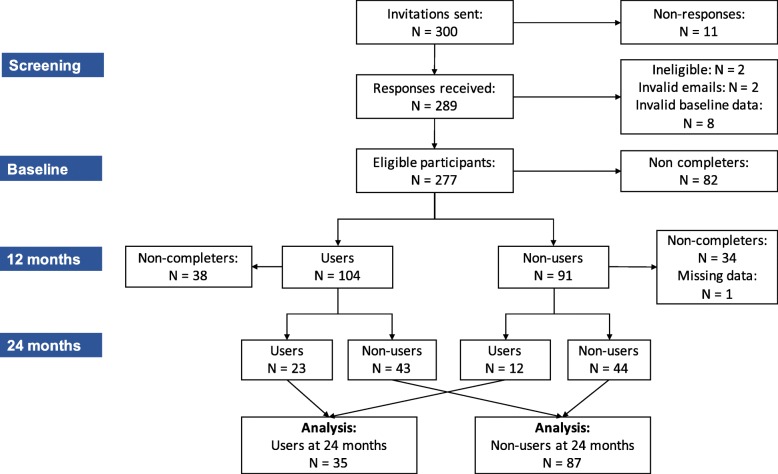
The study flowchart

Among the 300 invited participants, 289 participants completed the baseline questionnaires of which 12 participants were excluded due to missing/invalid data entry or not eligible (2 had no knee/hip pain and no previous diagnosed OA). A total of 277 people was included at baseline and 195 participants (70%) completed outcome measures at 12 months. At 24 months, we observed further attrition and received 122 completed responses (non-users: *n* = 87, users: *n* = 35) (Fig. 2). The response rate of 44% was much lower at this final 24-month follow up than at 12-months. Of the responders at 24 months (*n* = 122), 28% (n = 35) indicated they had used the updated website which included 12 new users (i.e. non-users at 12 months and users at 24 months) and 23 continuous users (i.e. users at both 12 and 24 months). Similar baseline demographics (e.g. age, gender, BMI and joint affected) were observed among non-users and non-completers at 24 months, but users at 24 months were slightly older, had lower BMI and more females than other groups (Table 1).

**Table 1:** Demographic characteristics of participants ^a^

Baseline characteristics	Users at 12 months	Non-users at 12 months	Non-completers at 12 months	Users at 24 months	Non-users at 24 months	Non-completers at 24 months
	*N* = 104	*N* = 91	*N* = 82	*N* = 35	*N* = 87	*N* = 73
Age (years)	60.5 (8.3)	60.9 (9.1)	61.6 (8.5)	62.4 (8.3)	60.3 (8.4)	60.3(9.3)
Female, *N* (%)	79 (76.0)	73 (80.2)	60 (73.2)	30 (85.7)	66 (75.0)	56 (77.8)
BMI (kg/m^2^)	30.4 (6.8)	32.5 (10.0)	30.4 (6.8)	29.9 (7.1)	31.6 (9.5)	31.8 (7.7)
Joint, N (%) ^b^						
Knee	54 (52.4)	38 (43.2)	45 (54.9)	19 (51.4)	42 (47.7)	32 (44.4)
Hip	20 (19.4)	16 (18.2)	7 (8.5)	5 (14.3)	18 (20.5)	13 (18.1)
Both	29 (28.2)	34 (38.6)	29 (35.4)	10 (28.6)	26 (29.5)	27 (37.5)

^a^Except where indicated otherwise, values are the mean (SD)

^b^Total percentage may not add up as it was possible to receive a risk of OA without indicating a joint

Abbreviation: *BMI* body mass index, *N* number, *SD* standard deviation

Within the user group, no significant changes were found in all the other domains of the HEIQ after adjustments for baseline age, sex and BMI. There were slight improvements in skill and techniques and constructive attitudes and approaches from 12 to 24 months. Non-users at 24 months showed slightly lower scores in all domains when subjected to the same analysis. There were improvements in skill and technique acquisition and less emotional distress in non-users but these changes were not statistically or clinically significant (Table 2). The comparisons between users and non-users at 24-months also showed no statistical and clinical significance in both univariable and multivariate models (Table 3). Only “emotional distress” score at 24 months was significantly higher in users (RR: 1.26, 95%CI: 0.11, 0.43), but became non-significant after adjusting for age, sex and BMI (Table 3). Excluding those crossed over from users to non-users over the 24-month period, a further sensitivity analysis was performed in people who were continuous users and non-users at 12 and 24 months showed similar results but even smaller differences (Additional file 1: Table S1).

**Table 2:** Within-group change from 12 to 24-month for each HEIQ domain in users and non-users of the updated ‘My Joint Pain’ website

HEIQ domains †	Users (*N* = 35)				Non-users (*N* = 87)			
	Baseline score	12 months score	24 months score	Within group difference from 12 to 24 months	Baseline score	12 months score	24 months score	Within group difference from 12 to 24 months
	mean (SD)	mean (SD)	mean (SD)	RR (95% CI) ^b^	mean (SD)	mean (SD)	mean (SD)	RR (95% CI) ^b^
1. Health-directed activity	2.92 (0.84)	2.99 (0.69)	3.00 (0.74)	1.000 (0.977, 1.023)	2.90 (0.71)	3.02 (0.71)	3.07 (0.78)	1.001 (0.987, 1.015)
2. Positive and active engagement in life	3.06 (0.45)	3.17 (0.48)	3.18 (0.56)	1.000 (0.978, 1.022)	3.06 (0.64)	3.10 (0.65)	3.23 (0.57)	1.003 (0.989, 1.017)
3. Emotional distress ^a^	2.34 (0.71)	2.10 (0.63)	2.22 (0.68)	1.002 (0.976, 1.028)	2.31 (0.71)	2.25 (0.72)	2.04 (0.74)	0.993 (0.976, 1.010)
4. Self-monitoring and insight	3.02 (0.41)	3.17 (0.43)	3.20 (0.46)	1.000 (0.979, 1.023)	3.09 (0.41)	3.13 (0.46)	3.22 (0.45)	1.002 (0.988, 1.016)
5. Constructive attitudes and approaches	3.06 (0.46)	3.06 (0.50)	3.19 (0.53)	1.003 (0.981, 1.026)	3.05 (0.53)	3.10 (0.60)	3.23 (0.54)	1.003 (0.989, 1.017)
6. Skill and technique acquisition	2.82 (0.43)	2.79 (0.44)	2.91 (0.53)	1.003 (0.980, 1.027)	2.72 (0.52)	2.84 (0.54)	3.05 (0.49)	1.006 (0.991, 1.020)
7. Social integration and support	2.54 (0.62)	2.74 (0.58)	2.79 (0.65)	1.000 (0.977, 1.024)	2.65 (0.52)	2.69 (0.60)	2.84 (0.53)	1.004 (0.989, 1.019)
8. Health service navigation	2.73 (0.57)	2.85 (0.53)	2.98 (0.58)	1.003 (0.980, 1.026)	2.94 (0.59)	2.98 (0.63)	3.06 (0.54)	1.002 (0.988, 1.016)

^a^Scores range from 1 to 4, a higher score indicates more favourable outcome except for emotional distress

^b^A positive mean difference indicates an improvement in outcomes except for emotional distress

Data in bold indicates variables with a *P* value < 0.05 for within-group comparisons

*HEIQ* health education impact questionnaire, *RR* risk ratio, *SD* standard deviation

**Table 3:** Differences of HEIQ scores between users and non-users of the updated ‘My Joint Pain’ website from 12 to 24 months

HeiQ domain ^a^	Univariable	Multivariable
	RR (95% CI)	RR (95% CI)
1. Health Directed Activity	0.969 (0.853, 1.101)	0.948 (0.760, 1.182)
2. Positive and Active Engagement in Life	0.889 (0.808, 0.979)	1.065 (0.853, 1.330)
3. Emotional Distress	**1.261 (1.113, 1.428)**	0.919 (0.733, 1.152)
4. Self-Monitoring and Insight	0.947 (0.867, 1.034)	1.110 (0.929, 1.325)
5. Constructive Attitudes and Approaches	0.997 (0.910, 1.092)	0.968 (0.788, 1.190)
6. Skill and Technique Acquisition	0.912 (0.823, 1.009)	0.840 (0.662, 1.068)
7. Social Integration and Support	0.911 (0.820, 1.011)	1.180 (0.957, 1.456)
8. Health Service Navigation	1.063 (0.952, 1.188)	1.100 (0.905, 1.336)

^a^Scores range from −3 to 3, a RR over 1 indicates a favourable effect in user group except for emotional distress. Multivariable models were adjusted for age, sex and body mass index

Data in bold indicates variables with a *P* value < 0.05 for between group comparisons

*HEIQ* Health Evaluation Impact Questionnaire, *RR* risk ratio, *CI* confidence interval

The results of OAQI pass rates showed higher pass rates (i.e. better quality of care) in “Treatment Alternatives” (12%), “Self-Management” (17%), “NSAIDs” (13%) and “Referral to Orthopaedic Surgeons” (23%) domains and lower pass rates in “Functional Assessment” (11%) and “Walking Aid Assessment” (7%) in users of the website comparing to non-users at 24 months (Table 4). A higher proportion of users experienced an improvement of OAQI from baseline to 24 months in “Acetaminophen (Paracetamol)” compared to non-users (23% versus 8%, *P* <  0.05), but due to very limited number of participants, the differences were not considered as clinically meaningful.

**Table 4:** The OAQI outcomes in various domains at baseline and 24 months for users and non-users of the updated ‘My Joint Pain’ website

		Users (*N* = 35)				Non-Users (*N* = 87)			
	Baseline	12 months	24 months	Improvement from 12 to 24 months	Baseline	12 months	24 Months	Improvement from 12 to 24 months	
	eligible N (QI pass rate) ^a^	eligible N (QI pass rate) ^a^	eligible N (QI pass rate) ^a^	N (%) ^a^	eligible N (QI pass rate) ^a^	eligible N (QI pass rate) ^a^	eligible N (QI pass rate) +	*N* (%) ^a^	*P* ^b^
1. Disease development	17 (53%)	21 (66%)	21 (62%)	4 (13%)	39 (50%)	42 (45%)	49 (63%)	13 (19%)	0.46
2. Treatment alternatives	13 (42%)	22 (75%)	24 (71%)	6 (18%)	52 (62%)	46 (54%)	51 (59%)	17 (20%)	0.91
3. Self-management	16 (47%)	17 (53%)	24 (71%)	5 (16%)	40 (48%)	47 (55%)	44 (54%)	9 (11%)	0.11
4. Lifestyle	16 (47%)	20 (61%)	21 (64%)	5 (16%)	43 (50%)	51 (59%)	52 (63%)	14 (17%)	0.88
5. Physical activity	29 (85%)	30 (86%)	31 (89%)	4 (11%)	63 (73%)	78 (90%)	71 (84%)	4 (4%)	0.24
6. Referral physical activity	22 (63%)	20 (59%)	22 (65%)	5 (15%)	39 (45%)	47 (55%)	50 (59%)	14 (17%)	0.98
7. Weight reduction	17 (85%)	15 (68%)	13 (65%)	1 (5%)	43 (70%)	41 (71%)	42 (70%)	6 (11%)	0.67
8. Referral weight reduction	1 (5%)	5 (28%)	4 (22%)	2 (13%)	9 (16%)	16 (29%)	14 (26%)	5 (10%)	0.89
9. Functional assessment	10 (33%)	16 (55%)	8 (36%)	1 (5%)	29 (42%)	31 (48%)	28 (47%)	7 (14%)	0.16
10. Walking aid assessment	5 (26%)	10 (40%)	5 (29%)	0 (0%)	10 (17%)	16 (30%)	17 (36%)	4 (10%)	0.11
11. Other aids assessment	4 (27%)	3 (14%)	3 (19%)	2 (14%)	5 (10%)	8 (17%)	7 (17%)	4 (11%)	0.86
12. Pain assessment	20 (57%)	17 (53%)	17 (50%)	6 (19%)	53 (62%)	50 (58%)	43 (51%)	9 (11%)	0.73
13. Acetaminophen (Paracetamol)	23 (66%)	21 (60%)	29 (83%)	8 (23%)	68 (78%)	70 (82%)	67 (78%)	7 (8%)	**< 0.05**
14. Stronger pain killers	16 (53%)	19 (63%)	17 (63%)	4 (15%)	48 (60%)	44 (59%)	40 (58%)	5 (8%)	0.80
15. NSAIDs	18 (75%)	21 (70%)	22 (85%)	4 (15%)	50 (76%)	44 (79%)	43 (72%)	4 (8%)	0.15
16. Cortisone	14 (48%)	9 (36%)	8 (36%)	2 (10%)	30 (48%)	24 (43%)	18 (32%)	3 (7%)	0.31
17. Referral to orthopaedic surgeon	12 (55%)	14 (67%)	14 (74%)	2 (11%)	29 (46%)	32 (52%)	28(51%)	5 (11%)	0.78

^a^Number of eligible answers calculated as total number of yes and no answers (whose who did answered “not applicable” or “do not remember” answers were excluded); pass rate is the proportion of yes answers over the total number of eligible answers

^a^Improvement in OAQI defined as those answered “no” at 12 months and answered “yes” at 24 months

^b^Data in bold indicates variables with a *P* value < 0.05 for changes in OAQI (negative, no change, positive) from 12 to 24 months between users and non-users

*OAQI* Osteoarthritis Quality Indicator; *NSAIDs* non-steroidal anti-inflammatory drugs

## Discussion

This study evaluated the effects of health impact and quality of care after using an updated version of ‘My Joint Pain’, a web-based platform for OA self-management education at 24 months compared to non-users. There were no significant changes in health education measuring by the HEIQ from 12 to 24 months in users of the website. Between-group (users versus non-users) differences failed to find statistically significant favourable changes in HEIQ, however, non-users seem to experience less emotional stress. Users showed higher improvements on several items in the OAQI, including appropriate information about self-management, treatment alternatives and the use of NSAIDs (effects and side-effects). However, outcomes from this study were of little clinical relevance given the small differences observed between groups and the increasing loss of volunteers throughout the study.

The findings of the 24-month evaluation of the ‘My Joint Pain’ website were similar to the 12-month evaluation improving knowledge and healthy behavioural changes especially self-management and pharmacological treatments [23]. These findings reinforce the concept that the website aids in promoting self-management and non-surgical, non-pharmacological treatments which are the first-line recommendations [30].

An unexpected finding is the deterioration of emotional distress at 24 months in the users compared to non-users. The reason for the worsening emotional wellbeing could be attributed to the natural history of the disease that continuously progresses over time. Evidence also suggests people with fewer symptoms are more likely to drop out in both clinical and research settings [31], and those who needed more support or had more severe symptom tend to complete follow-ups.

The ‘My Joint Pain’ website addressed several barriers to self-management, such as financial limitations and lack of personalised recommendations by providing free resources with comprehensive assessment instruments to tailor strategies [32]. From the substantial reduction in the number of continuous users over time, it suggests that there is still a lack of a broader endorsement that allows healthcare professionals to encourage their patients to use the website. This has been recognised as a key barrier for the uptake of self-management and education resources [33]. However, this approach has challenges given that the attitudes and beliefs of primary care providers, especially general practitioners (GPs), are not necessarily supportive of recommended evidence-based treatments such as exercise [34, 35]. This might be due to the fact that the role of GPs in initiating exercise for OA management was not outlined in guideline recommendations [36, 37] and there is no explicit expectation about whether GPs should refer patients for exercise therapies, advise general or specific exercises or prescribe exercises. Therefore, future work is also needed to identify the optimal means of supporting and educating GPs at the clinical, educational and service level, to improve certainty and confidence about the value of recommended care and to use them in practice. Importantly, to increase its adoption and uptake by consumers and health professionals, it is critical to embed an optimised platform into routine clinical practice and develop more interactive functions to facilitate shared clinical decision-making between patients and doctors.

As an emerging area, there is still room for improvement in the design of current Internet-based health interventions. Take the example of ‘My Joint Pain’ website, it provided a personalised treatment plan for the users, but has not adequately provided social support and contact with health professionals through the platform [38]. When these pillars have been addressed in other rheumatic online interventions, improvements in quality of life and global health scores were observed especially in studies that have interactive online forums and web-conferences [39–42]. Currently, the website is English-focused which is unable to match the increasing language and literacy needs in a growingly multicultural society with over 300 languages spoken in Australian homes [43, 44]. Similar resources in the future should include content that addresses health literacy and language barrier with the inclusion of consumer assisted content reviews and multilingual resources [45]. Additionally, to maintain relevance, the online platform should be continuously updated with the latest recommendations modified to meet the needs and regulations of the local osteoarthritic community.

Several limitations of the evaluation study were identified. A critical problem was the substantial dropout of participants over the 24 months. With over 56% of the baseline population not responding at 24 months, group sizes were vastly different and below the required sample size. The attrition of volunteers also prevented further evaluation by stratified analyses into subgroups based on gender or age. The low response rate was also hampered by the limitation of the contact information collected at baseline being restricted to electronic email addresses. The lack of other contact information prevented the possibility of following other communication methods to address the lack of response. In addition, the study did not collect information about population defining characteristics such as depression and anxiety status or evaluations of the quality of life, which would have been valuable in contrast to other studies. A caveat to the use of quasi-experimental study design was the inability to randomise participants, and more crucially in a study with several time points, it was not possible to maintain grouping for the whole study. This limitation resulted in several different groups with varying degrees of usage of both the old and updated websites which could not be assessed given the small sample size. In addition, the study survey was designed as a self-reported indicator of usage limited to “Yes” or “No”. As a result, we were unable to ascertain a dose-effect of the website. Quantitative web statistics such as the number of secure logins and time spent should be collected which will be helpful for future evaluation and optimisation. Findings from this latest evaluation indicate a need for better-conducted and well-designed studies such as RCTs to efficiently evaluate the use of an online intervention.

## Conclusion

Web-based healthcare resources are increasingly utilised as a response to the overburdened health system and an ageing population. The 24-month evaluation of the ‘My Joint Pain’ website didn’t find significant improvement in terms of health education, but it may help to deliver useful information on self-management and appropriate use of pharmacological treatments. A large number of drop-outs at 24 months suggested that there is a need to increase its adoption and uptake by consumers and health professionals. Additional research with a larger sample size would be helpful to evaluate the cost-effectiveness of this web-based intervention further. More strategies are also needed to facilitate the process of implementing evidence-based OA self-management and education resources into current practice.

## Supplementary information

**Additional file 1: Table S1.** Within-group change from 12 to 24-month for each HEIQ domain in continuous users and non-users of the ‘My Joint Pain’ website from 12 to 24 months.

## Data Availability

The datasets used and/or analysed during the current study are available from the corresponding author on reasonable request.

## Abbreviations

BMI: Body mass index
GP: General practitioner
HEIQ: Health Evaluation Impact Questionnaire
HON: Health on the Net
IPDAS: International Patient Decision Aid Standards
NHMRC: Australia’s National Health and Medical Research Council
NSAID: Non-steroidal anti-inflammatory drug
OA: Osteoarthritis
OAQI: Osteoarthritis Quality Indicator
