# Supplementary material for: My joint pain, a web-based resource, effects on education and quality of care at 24 months
Source: BMC Musculoskelet Disord. 2020 Feb 6;21:79. doi: 10.1186/s12891-020-3074-2 (PMC7006132; doi:10.1186/s12891-020-3074-2)
Supplement: Supplementary file 1 — Additional file 1: Table S1. Within-group change from 12 to 24-month for each HEIQ domain in continuous users and non-users of the ‘My Joint Pain’ website from 12 to 24 months. [file 12891_2020_3074_MOESM1_ESM.docx]

Table S1. Within-group change from 12 to 24-month for each HEIQ domain in continuous users and non-users of the ‘My Joint Pain’ website from 12 to 24 months.

| HEIQ domains † | Continuous Users (N = 23) | | | |  | Continuous Non-users (N = 44) | | | |
| --- | --- | --- | --- | --- | --- | --- | --- | --- | --- |
|  | Baseline score | 12 months score | 24 months score | Within group difference from 12 to 24 months |  | Baseline score | 12 months score | 24 months score | Within group difference from 12 to 24 months |
|  | mean (SD) | mean (SD) | mean (SD) | RR (95% CI) †† |  | mean (SD) | mean (SD) | mean (SD) | RR (95% CI) †† |
| 1. Health-directed activity | 2.84 (0.94) | 2.87 (0.70) | 2.93 (0.78) | 1.002 (0.983, 1.022) |  | 3.09 (0.66) | 3.10 (0.69) | 3.22 (0.74) | 1.001 (0.973, 1.030) |
| 2. Positive and active engagement in life | 3.03 (0.41) | 3.03 (0.47) | 3.16 (0.53) | 1.003 (0.984, 1.023) |  | 3.21 (0.58) | 3.15 (0.67) | 3.31 (0.56) | 1.003 (0.975, 1.031) |
| 3. Emotional distress † | 2.40 (0.62) | 2.32 (0.50) | 2.25 (0.60) | 0.996 (0.972, 1.020) |  | 2.28 (0.79) | 2.19 (0.81) | 2.02 (0.82) | 0.999 (0.968, 1.032) |
| 4. Self-monitoring and insight | 3.01 (0.42) | 3.07 (0.33) | 3.14 (0.46) | 1.002 (0.983, 1.022) |  | 3.14 (0.47) | 3.16 (0.53) | 3.27 (0.48) | 1.002 (0.974, 1.029) |
| 5. Constructive attitudes and approaches | 3.05 (0.44) | 2.98 (0.48) | 3.15 (0.58) | 1.002 (0.982, 1.021) |  | 3.21 (0.53) | 3.16 (0.66) | 3.26 (0.59) | 1.004 (0.976, 1.032) |
| 6. Skill and technique acquisition | 2.76 (0.32) | 2.79 (0.37) | 2.86 (0.48) | 1.005 (0.985, 1.026) |  | 2.78 (0.53) | 2.87 (0.61) | 3.09 (0.53) | 1.001 (0.973, 1.030) |
| 7. Social integration and support | 2.63 (0.58) | 2.72 (0.52) | 2.82 (0.65) | 1.005 (0.984, 1.027) |  | 2.65 (0.61) | 2.60 (0.72) | 2.81 (0.60) | 1.001 (0.973, 1.031) |
| 8. Health service navigation | 2.69 (0.45) | 2.82 (0.46) | 3.02 (0.57) | 1.001 (0.982, 1.021) |  | 3.03 (0.62) | 3.04 (0.75) | 3.12 (0.64) | 1.004 (0.976, 1.033) |

† Higher score indicates more favourable outcome except for emotional distress.

†† A positive mean difference indicates an improvement in outcomes except for emotional distress.

Data in bold indicates variables with a *P* value < 0.05 for within-group comparisons.

HEIQ, health education impact questionnaire; RR, risk ratio; SD, standard deviation.
